# Supplementary material for: Estimating the impact of interventions against COVID-19: From lockdown to vaccination
Source: PLoS One. 2021 Dec 17;16(12):e0261330. doi: 10.1371/journal.pone.0261330 (PMC8683038; doi:10.1371/journal.pone.0261330)
Supplement: Attachment — Submitted filename: Response to Reviewers.pdf [file pone.0261330.s001.pdf]

Response to Reviewers

Manuscript Number: PONE-D-21-09294

My co-author and I welcome the comments on our paper 'Estimating the impact of interventions against COVID-19: from lockdown to vaccination'. We appreciate the thorough and insightful quality of the reviews and have improved our article accordingly, with substantial revisions.

We have improved the description of the model environment and how the agents interact in it, and released the code for our model in a public repository:

`https://github.com/abm-covid-lux/abmlux`.

All input and output data, underlying the findings in the manuscript, are now fully available. The input data can be found in the above repository, while the output data, used to create the plots and tables in the manuscript, is located in a separate public repository:

`https://github.com/abm-covid-lux/output`.

We have now made clear how the sensitivity analysis was carried out and reported the outcome of this analysis. We have performed 790 additional simulations, on top of the 290 performed for the original manuscript, and included additional statistical analysis.

Detailed responses to each comment raised in the review are as follows, starting with the journal requirements and followed by those of the two reviewers:

Journal Requirements

1. 'Please ensure that your manuscript meets PLOS ONE's style requirements, including those for file naming.'

Response: We believe that these requirements are met, however we welcome any remarks to the contrary. We have amended the appearance of several tables in case their appearance was in conflict with the style requirements of the journal.

2. 'In your Methods section, please ensure that sufficient information to make the study reproducible are provided (for example, by reporting the equations representing the model, and describing parameters and assumptions applied).'

Response: Sufficient information to make the study reproducible has now been provided, with all code and input data publicly available on GitHub. As now explained in the manuscript, the full parametrization for each scenario can be found in a corresponding `config.yaml` file. These configuration files are located in the repositories, the default configuration being found in the `Scenarios/Luxembourg` folder of the `abmlux` repository. In addition, the methods section now reports several of the key equations appearing in the model, describing more clearly and precisely how agents choose locations and potentially infect one another.

3. 'We note that you have indicated that data from this study are available upon request. PLOS only allows data to be available upon request if there are legal or ethical restrictions on sharing data publicly. If there are ethical or legal restrictions on sharing a de-identified data set, please explain them in detail. If there are no restrictions, please upload the minimal anonymized data set necessary to replicate your study findings as either Supporting Information files or to a stable, public repository and provide us with the relevant URLs, DOIs, or accession numbers.'

Response: All data can now be found, freely accessible, in the above repositories. All input data can be found in the `Scenarios/Luxembourg` folder of the `abmlux` repository while all output data can be found in the `output` repository. Anonymized data from the 2014 Luxembourg Time-Use Survey, for example, can now be found alongside the other input data. We have received personal communication from STATEC, the government statistics service of Luxembourg, giving permission for this data to be published under the Creative Commons Attribution License (CCAL) CC BY 4.0.

4. 'Please note that in order to use the direct billing option the corresponding author must be affiliated with the chosen institute. Please either amend your manuscript to change the affiliation or corresponding author, or email us at plosone@plos.org with a request to remove this option.'

Response: All publications funded by the FNR, the Luxembourg National Research Fund, are paid via Chronos Hub. Therefore, after the FNR checks our eligibility for the open access fee refund, which typically takes place once an article is accepted for publication, the invoice address can be specified. On the advice of the University of Luxembourg, we therefore request your input on removing or not the direct billing option.

5. 'Please include both an updated Funding Statement and Competing Interests Statement in your cover letter.'

Response. This project was funded by a grant from the COVID-19 Fast-Track program of the Fonds National de la Recherche Luxembourg (`https://www.fnr.lu/`), with the grant being awarded to Dr. J. Thompson. The reference number for this project is:

COVID-19/2020-2/14858807/ABMLUX

The funder provided support in the form of salary for Dr. S. Wattam, but did not have any role in the study design, data collection and analysis, decision to publish, or preparation of the manuscript. The specific roles of authors are articulated in the 'Author Contributions' section. One of the authors, Dr. S. Wattam, is affiliated with a commercial company, W&P Academic Consultancy, but we declare that no competing interests resulted from this. In particular, this affiliation does not alter our adherence to PLOS ONE policies on sharing data and materials.

6. 'We note you have included a table to which you do not refer in the text of your manuscript. Please ensure that you refer to Table 5 in your text.'

Response: Table 5 has now been referred to in the text.

7. 'We note that Figure 2 in your submission contain map images which may be copyrighted.'

Response: The shapefile used to create Figure 2 was shared by the Government of Luxembourg under a CC0 1.0 Universal (CC0 1.0) Public Domain Dedication license:

`https://data.public.lu/en/datasets/limites-administratives-du-grand-duche-de-luxembourg/`

A reference to this url has now been included in the manuscript, before Fig 2.

Reviewer #1

1. 'The paper discussed the sensitivity of the number of dead to the distribution of care homes, and the sensitivity of our model with respect to the probability of being asymptomatic and the transmission probability. Which parameters is each model outcome sensitive to? Which approach did the authors use to perform sensitivity analysis (e.g. univariate, multivariate, or probabilistic)? I suggest the authors to include a table that

summarizes the values of model parameters in the baseline scenario and the distributions used in sensitivity analysis.'

Response: We have added a subsection on Sensitivity Analysis to end of the Model Evaluation section. The analysis performed was univariate. We estimated the partial derivatives of total deaths with respect to the probability of being asymptomatic, the transmission probability, and also the total number of care homes. We have plotted the relevant mappings, using output data from a large number of additional simulations. These plots illustrate that our model is not highly sensitive to independent variations in these parameters. Therefore, since the primary objective of this article is to compare interventions against the baseline scenario, with the interventions being implemented on top of the baseline model in a very natural way, we do not expect our final conclusions to be highly sensitive to small variations in these parameters. A more sophisticated analysis is currently out of reach, due to the long runtime of the model.

2. 'How was the vaccine efficacy implemented in the model not very clear? Did the authors refer to the vaccine efficacy in this paper as efficacy against confirmed Covid-19 case or efficacy against transmission? What state variables of agents were affected if they are vaccinated? (Did the vaccinated agents have their state of infection changed from Susceptible to Recovered/Immune at the probability p1 and p2?) How did vaccination affect the transmission if susceptible vaccinated individuals come into contact with infected individuals and their disease progression if infected?'

Response: The subsection on vaccination, appearing in the methods section, has now been rewritten to make this all much clearer. Efficacy in our model refers to the probability of being protected against infection, after receiving a dose of the vaccine. The health of protected individuals is blocked from making the state transition **Susceptible** to **Exposed**. Protected individuals are neither able to catch the virus nor transmit it, unless they are infected at the time of vaccination in which case they are still able to transmit it.

3. 'The number of simulations (ten) performed for each scenario seem quite small for a highly stochastic model like this model. I appreciate the extensive runtime for this high-resolution agent-based model. However, how did you decide that 10 simulations were sufficient to capture the true behaviours of the system? Would reducing the resolution and increasing the number of simulations produce outcomes with less uncertainty?'

Response: We have now increased the number of simulations for the baseline scenario from 10 to 100, as illustrated in Fig 17 and Fig 18, with several hundred additional simulations added to other subsections. The output distributions are concentrated around the mean and the sample variance is low, with the variance being much higher for low-resolution scaled versions of the model. This is a feature of the model that we observed early in the project, so given constraints on both time and resources, we decided on a small number of high resolution simulations, as opposed to a large number of low resolution simulations. Moreover, simulating with a high time resolution was necessary to properly implement the interventions and to capture brief encounters taking place outside the home, work or school.

4. 'I appreciate that it is a very sophisticated and detailed model. However, would the purposes of the study be achieved with a simpler model such as a stochastic compartment model or an ABM with simpler structure? The authors mentioned that some of their results were similar to results from other studies which using simpler models. In this case, what would be the justification for building a more complex model?'

Response: Some of the purposes of the study could have been achieved with a simpler model, however only a detailed agent-based model is capable of supporting the full range of scenarios that we wanted to investigate. As the pandemic unfolded, the detail in the model was useful, and this shaped some of the design. With a stochastic compartmental model, or

a simpler ABM, it would have been much more difficult to model all of the interventions simultaneously and realistically. The structure of our code is actually quite simple, and allows for the interventions to be modelled in a transparent and intuitive way. For example, instead of representing a lockdown using a reduction in a contact rate parameter, as is done in some models, in our model a lockdown simply directs agents home, which is more intuitive. Moreover, our model is able to capture a much higher level of heterogeneity than would be possible using a simpler compartmental model. This is an important point since these heterogeneities result in significant differences in output, as shown in Fig 19 and Table 8 of our revised manuscript.

Reviewer #2

1. 'The overall text is well written but sometimes exaggerates in length, and many paragraphs could be replaced by a diagram, a table, etc, reducing the length of the text and yet improving the readability of the results.'

Response: Several paragraphs have now been reduced in length, and some omitted altogether. We have introduced a number of additional tables, as suggested, in particular Tables 7, 8, 9, 10 and 11. We agree that the text was in places rather verbose, so several paragraphs have been rewritten more concisely.

2. 'There is a problem with the location and quality of the images in the review text that harmed the reading and understanding of the text. All images appeared at the end of the text, which is inopportune. I suggest fixing this issue for the next review rounds.'

Response: The images appeared at the end of the text to meet the requirements of the journal, however it does make the article difficult to read, so in the revised version we have reinserted the images directly into the text. This should also avoid any loss in image quality, which we suspect occurred during the image submission process. Moreover, we have converted our figure files using the PACE digital diagnostic tool, as suggested by the editor.

3. 'In the State of Art section I think is missing a lot of recent machine learning and ABM models. This section can be improved with a deeper revision of recent research of agent-based epidemiological simulation of COVID-19.'

Response: We have now performed a much deeper review of relevant literature and have essentially rewritten the State of Art section. We now refer to a much broader range of literature, including many agent-based models of COVID-19 not referred to at all in the original version, and have inserted a paragraph specifically focussed on machine learning and epidemic modelling of COVID-19. Overall, 49 new references have been added to the manuscript.

4. 'In the Methods section, the authors focused on the semantic meaning of the system components, which is perfectly understandable given the scope of the research. However, a little bit more details about the software implementation could improve the text and help the readers interested in implement or even employ the model. On the other hand, some descriptions of the model behavior (e. g. the school and classes descriptions) could be abbreviated. Or you simply share the link of the source code or you should remove from the text the promise of opening the code in the future.'

Response: Our code is now freely accessible on GitHub, together with all the documentation and input data required for other users to run the simulations. In addition, we have added some equations to the Methods section to help clarify the exposition, while abbreviating some other paragraphs, including the school and classroom descriptions.

5. 'The authors let it clear in the text that the ABM has a network structure. But the Locations subsection gives the impression that the software is grid-oriented. How explicitly

occur the relationships between the locations and the agents? How are they represented? Along with the text, the authors recovered the notion of a network model but in a fashion hard to understand. A good example is a car using by the agents. There will be a unique car location shared by all agents? I think that including a Network Diagram in this section, showing a small sample of the agents and their interactions will help the readers to understand better how your model works.'

Response: We agree that the talk of networks was somewhat misleading, so we have rewritten parts of the Introduction and the Locations subsection accordingly. In particular, unlike some agent-based models, our model is not based on networks. The Spatial Distribution and Location Choice subsections have also been rewritten, to help clarify the relationship between agents and locations and the role played by the grid (when initializing the random environment, locations are assigned coordinates to a resolution of 1m, first by selecting a 1km grid square and then by sampling uniformly within that square, with this being the only role played by the grid). To address the final point about network diagrams, we have now included a new figure, Fig 7, which for three randomly selected agents plots their home and personal locations on the map of the region. The line segments in Fig 7 simply illustrate the distance between an agent's home and the other locations, the agents being able to travel between these locations in any order.

6. 'The ODD protocol in Appendix has an interesting high-level description of the whole system. However, I still miss some logical diagrams of the software structure and architecture (maybe a UML package diagram, or a component diagram ?)'

Response: We hope that the releasing of our code in a documented repository satisfies this requirement. We can provide a UML package diagram or a component diagram if required, possibly in exchange for relaxation of any length limits.

7. 'In Model Evaluation and Validation, much of these paragraphs can be simplified or synthesized using mathematical formulas or replaced by a table with the parameters and their sources.'

Response: This section was excessively wordy in places, so has now been simplified with some paragraphs removed entirely.

8. 'The results are very interesting but are spread in text and should be synthesized in a table, with the results of the proposed model and the SEIR model side by side, together with a statistical significance test of the difference between them.'

Response: We have addressed this comment with Tables 7, 8, 9, 10 and 11. Table 8 puts the results of the two models side by side, together with a statistical significance test of the difference between them, as requested.

Having made these revisions, carefully addressing each of the helpful comments raised in the review, we feel that the quality of our manuscript has been substantially improved and present the revised version for your consideration.

Yours sincerely,

James Thompson
