# Supplementary material for: Estimating the impact of interventions against COVID-19: From lockdown to vaccination
Source: PLoS One. 2021 Dec 17;16(12):e0261330. doi: 10.1371/journal.pone.0261330 (PMC8683038; doi:10.1371/journal.pone.0261330)
Supplement: Attachment — Submitted filename: Response to Reviewers_2.pdf [file pone.0261330.s002.pdf]

Response to Reviewers

Manuscript Number: PONE-D-21-09294

My co-author and I welcome the latest comments on our paper ‘Estimating the impact of interventions against COVID-19: from lockdown to vaccination’ and have made appropriate revisions. Our responses to the comments raised in the review are as follows:

Reviewer #1

1. ‘Regarding the vaccination efficacy, the authors only consider the all-or-nothing scenario that appears not to be the case for COVID-19 vaccines. Experiments that consider the efficacy in terms of both infection and transmission would be more useful.’

Response: Our model of vaccination, despite taking into account such factors as vaccine hesitancy, limits on daily doses, a delay between first and second doses, and the order in which individuals are vaccinated, does indeed implement vaccine efficacy in a relatively simple way. Immune responses are in reality very diverse and complex, and vary significantly between individuals, with some individuals being unable to mount an immune response at all. We therefore agree that it would be extremely useful to develop a more detailed model of immunity, and implement it in the simulations. For this reason, we are currently working on a project that aims to do just that, using a model that distinguishes immunity to infection from immunity to transmission, as suggested by the reviewer, and also immunity against symptomatic illness or severe disease. This new project also considers factors such as partial immunity, waning immunity, cross immunity for multiple variants and multiple vaccines. We feel that a deeper exploration of the topic of COVID-19 vaccination and immunity requires its own project, with many of the aforementioned factors being beyond the scope of the submitted manuscript. The all-or-nothing scenario is assumed for simplicity, with this assumption often being found in the literature. We have nonetheless revised the conclusion to our manuscript to emphasise, in response to the reviewer’s comment, that the all-or-nothing approach is a limitation of the study.

2. ‘I found the justification on the appropriateness of agent-based models compared with other types of stochastic compartment models still not persuasive. If the intention of the model is to compare the relative effectiveness between interventions and the authors predict that the conclusions are not highly sensitive to parameter changes, how would adding more heterogeneities into the model would result in significant differences in output. Also, if a sophisticated analysis is out of reach due to the long runtime of the model, adding more heterogeneities means adding more uncertainty into the model without being able to manage such uncertainty. This sounds like a disadvantage of ABM.’

Response: While it is true that stochastic compartmental models have some advantages, agent-based models reflect the fact that epidemics do in fact result from the actions and interactions of individuals, offering a natural ’bottom-up’ methodology to complement the ’top-down’ methodology of the equation-based compartmental models.

Our model features heterogeneous behaviour and age, since these are clearly important factors in determining the outcome of a COVID-19 epidemic, but on the other hand does not distinguish agents by sex or by the presence of underlying medical conditions, and keeps sub-models of public transport and leisure relatively simple. Overall, our aim was to find a reasonable balance between detail and flexibility.

An agent-based model need not have more parameter uncertainty than a compartmental model, since many of the features that require parametrizing in a compartmental model, for

example the flows of population between locations, are emergent in the agent-based model, consequences of the rules imposed on agents rather than the values assigned to certain variables. In an agent-based model, these rules are often intuitive, allowing for realistic representations of policy interventions. For example, in the agent-based model, lockdowns simply direct agents to stay at home (unless they are exempt), while in a compartmental model, a lockdown is represented more abstractly as a reduction in β, the contact rate multiplied by the transmission probability. This reduction in β then requires its own estimate, a step not required in the agent-based model. Agent-based models, when implemented efficiently, can actually reduce uncertainty when compared to a heavily parametrized compartmental model.

Moreover, if it turns out that certain additional heterogeneities really do not have a big impact on the outcome, then this is in itself an interesting result, that might be difficult to obtain using a stochastic compartmental model.

We should point out that, in the case of our model, the limiting factor is not so much the complexity of the model, but rather the fact that it is written in Python, which executes relatively slowly. For this reason, we are currently working on performance enhancements that will reduce the runtime from several hours to several minutes, ultimately allowing for a more sophisticated statistical analysis of uncertainty. This is, however, still a work in progress.

We have revised our manuscript to include further discussion of the limitations of the modelling approach, and how we intend to address them in the future.

Having made these revisions, addressing the comments raised in the review, we feel that the quality of our manuscript has improved and we present the revised version for your consideration.

Yours sincerely,

James Thompson
